# The p53-induced Siva-1 plays a significant role in cisplatin-mediated apoptosis

**DOI:** 10.4103/1477-3163.45389

**Published:** 2009-02-07

**Authors:** John L. Barkinge, Radhika Gudi, Hawkins Sarah, Fei Chu, Alip Borthakur, Bellur S. Prabhakar, Kanteti V.S. Prasad

**Affiliations:** Department of Microbiology and Immunology, University of Illinois at Chicago, 835 S. Wolcott Ave., MC 790, Chicago, IL 60612, USA; 1 Children's Memorial Hospital, Northwestern University, Chicago, IL, 60614, USA; 2 Department of Medicine, University of Illinois at Chicago, 60612, USA

**Keywords:** Apoptosis, Bcl-2 family, cisplatin, cytochrome c, p53, Siva-1

## Abstract

**Background:**

The pro-apoptotic protein Siva-1 functions in both extrinsic and intrinsic cell death signaling; however, the exact contribution of the endogenous Siva-1 to DNA damage-induced apoptosis is unclear. Using cisplatin, a chemotherapeutic drug, to induce DNA damage and cell death, we determined the role of Siva-1.

**Methods:**

Cisplatin treated HCT116 colorectal carcinoma cells (p53+/+ and -/-) were used in the study. With the help of recombinant lentivirus that can express siSiva (siRNA that specifically targets Siva-1), we also generated Siva-1 knockdown HCT116 cells. Apoptosis was determined by tetramethyl rhodamine methyl ester (TMRM) staining and propidium iodide (PI) staining.

**Results:**

Treatment with cisplatin induced Siva-1 expression in a p53 dependent manner. In Siva-1 knockdown p53+/+ HCT116 colorectal carcinoma cells, loss of Siva-1 expression conferred significant resistance to cisplatin-induced apoptosis. Although Siva-1 levels were positively regulated by p53, Siva-1-induced apoptosis did not require p53. Despite the fact that Siva-1 lacks even a minimal BH3 domain, similar to other proapoptotic Bcl2 family members induced by p53, we showed that Siva-1 mediated apoptosis is characterized by Bax oligomerization and cytochrome c leakage from mitochondria. The putative amphipathic helical region in Siva-1 (SAH) appeared to function analogously to a BH3 domain.

**Conclusion:**

The p53 induced Siva-1 is one of the effector molecules, which plays a significant role in DNA damage-induced cell death.

## Background

It is estimated that at least 50% of human cancers can be attributed to the ablation or dysfunction of the p53 gene.[1] As a transcription factor, p53 has been implicated in the regulation of numerous tumor suppressor genes. In cases of irreparable DNA damage, p53 is well-characterized by its ability to transcriptionally upregulate various Bcl-2 family target genes involved in apoptosis.[2] Studies have also identified genes outside the Bcl-2 family, which facilitate apoptosis and are regulated by p53.[3]. Among these, the Siva-1 gene is transcriptionally induced by p53, at levels exceeding that of Bax, in response to genotoxic stress.[4] Despite a growing understanding of the role of p53 in DNA damage response pathways, the exact function of many of its unique target genes remains to be elucidated.

Siva-1 signaling output influences both extrinsic and intrinsic apoptotic pathways.[5–10] DNA microarray analyses have demonstrated enhanced Siva-1 activity in response to chemotherapeutic agents in several transformed cell lines.[4,11–12] Further, we have shown that Siva-1 can act synergistically with cisplatin and promote apoptosis in breast cancer cell lines, regardless of the p53 or Bcl-2 status.[13] This finding correlates with our previous observations of direct inhibition of Bcl-2 and Bcl-x_L_ by Siva-1, in response to genotoxic stress.[8–9] In the current study, we examined the role of Siva-1 in cisplatin-mediated apoptosis in HCT116 p53 +/+ and p53-/-cells. Using siRNA to abrogate endogenous Siva-1 expression, we demonstrate the importance of its role in DNA damage-induced apoptosis in p53 WT cells. In addition, we show that expression of Siva-1 can promote apoptosis, even in cells devoid of p53 function. Based on its ability to interact with Bcl-2 and Bcl-x_L_ and to promote Bax oligomerization, we further propose that Siva-1 functions like a BH- only pro-apoptotic member of the Bcl-2 family.

## Materials and Methods

### Cell lines and Reagents

Colon cancer cells HCT116 p53+/+ and p53-/- (kind gifts of Dr. Bert Vogelstein, Johns Hopkins University,)) were cultured in McCoy's 5A medium, supplemented with 10% FCS, 1 mM sodium pyruvate, 1 mM HEPES buffer, and 100 U/mL antibiotic/antimycotic (Gibco, Invitrogen). Human T cell lymphoma Jurkat and MDA MB-231 human breast cancer cells were maintained with RPMI medium.

The chemotherapeutic reagent cisplatin was purchased from Sigma and prepared in dimethyl sulfoxide (DMSO); UV radiation doses were applied via an XL-1000 Spectrolinker (Spectronics Corp.) UV crosslinker.

The following antibodies were purchased from companies that have been indicated; respective dilutions are also indicated for those used in western blotting analysis or intracellular staining: mouse monoclonal β actin (Sigma, 1:2,000); rabbit polyclonal caspase-9 (p-10) and Bax (Santa Cruz, 1:500); rabbit polyclonal Bcl-xL, Bcl-2, and green fluorescence protein (GFP) (Santa Cruz, 1:1,000); mouse monoclonal GST, Bax, Hemagglutinin (HA), and c-myc (Santa Cruz, 1:1,000); mouse monoclonalcytochrome C (BD Biosciences, 1:1,000); mouse monoclonal cytochrome C oxidase IV (COX IV) (Molecular Probes, Invitrogen, 1:1,000); Bax 6A7 (BD Biosciences); mouse monoclonal p53 (Cell Signaling Technologies, 1:1,000); rabbit IgG-HRP and mouse IgGHRP (Santa Cruz, 1:10,000); mouse IgG-HRP Trublot (Ebiosciences, 1:1,000); and mouse IgG-FITC (Life Source Technologies, 1:200). The Siva-1 antibody was generated commercially in rabbits, via injection of a previously described recombinant Siva-1 protein, expressed in and isolated from BL21 E. *coli* strains [14]. Rabbit antiserum was further purified using Thiophilic Resin (Clontech, 1:1,000), supplemented as above (Gibco).

### Recombinant Lentiviruses

Generation of recombinant lentiviruses encoding either a scrambled control or unique shRNA sequences targeted against Siva-1 has been previously described.[10] HCT116 p53+/+ and Jurkat cells were infected with equivalent titers of control and Siva-1 siRNA-producing viruses were applied to cells that were subsequently incubated for at least 48 hours prior to each experiment. The cells were further sorted on the basis of GFP expression (MoFlo High Speed Cell Sorter). The GFP expression after cell sorting was greater than 90%. The cell morphology and growth remained unchanged, indicating negligible cytotoxic effects (data not shown). Knockdown of endogenous Siva-1 was verified by western blotting with Siva-1 antibody. Lentiviral stocks were propagated in 293T cells via calcium phosphate co-transfection of pNLSIN (Siva-1 siRNA or Scrambled siRNA), pcTAT, pcRev, and pHit/G plasmids. Transfections were performed in the evening and the cell culture media was changed the following morning; 24 hours later, the media containing viral stocks were collected, spun down at 4,000 RPM in a table-top centrifuge to clear cellular debris, and used directly for infection or stored at 20°C

### Cell lysates and Inmmunoblotting

Preparation of whole cell lysates and various immunoblotting procedures were done according to previously published procedures.[10–12,15]

### Apoptosis

The percentage of cells undergoing apoptosis was determined by propidium iodide analysis of apoptotic DNA (Sub-G1 peak), as well as tetramethylrhodamine ester (TMRM) staining of Jurkat cells, as has been described.[8–10] Apoptosis was quantified by flow cytometry, on the basis of high GFP expression (siRNA positive cells), low 7AAD staining and high TMRM patterns. TMRM negative cells fitting these parameters were considered apoptotic.

### Subcellular Fractionation

This procedure was adapted from previously described protocols.[15–20] HCT116 p53-/- cells were harvested with corresponding media using cell scrapers and centrifuged at 200 × g (4°C) for five minutes. The resulting pellet was washed with PBS (pH 7.4) and spun down, again under the same conditions. Cells were resuspended in hypotonic buffer (20 mM Hepes, pH 7.5, 10 mM KCl, 1.5 mM MgCl2, 1 mM EDTA, 1 mM ethylene glycol tetraacetic acid (EGTA), protease inhibitor cocktail, 1X (Roche)) and incubated on ice for 10 minutes. They were lysed in a Dounce homogenizer by applying 50 strokes with a type B pestle. Hypotonic buffer containing 1M sucrose was added to the lysates, to obtain a final sucrose concentration of 250 mM. Whole cell lysate samples from the suspension were set aside and the remaining volumes were centrifuged at 750 × g, to separate intact cells and nuclei. The resulting pellets were discarded and the supernatants were centrifuged further at 10,000 × g for 10 minutes, to obtain heavy membrane (HM, mitochondria-enriched) fractions. The supernatants were saved as the S-100/LM fractions and the HM fraction pellets lysed in NP40 buffer (20 mM Tris, pH 7.4, 150 mM NaCl, 1% NP40, v/v, 0.1% sodium deoxycholate) for western blotting analysis. MCF-7 mitochondria were prepared similarly, but disrupted by nitrogen cavitation.

### Chemical Cross-linking

In order to observe Bax oligomers, MDA-MB-231 and HCT116 cell lysates were prepared by fractionation and subsequently incubated with 10 mM bis-maleimide hexane (BMH, Pierce) or DMSO (vehicle control), in the dark for 30 minutes, at room temperature.[15] The reaction was quenched by adding 10 mM β-mercaptoethanol. Samples were prepared for western blotting, as previously indicated and oligomerization was determined by molecular weight analysis.

## Results

### Cisplatin-induced Siva-1 expression is p53 dependent

**Figure 1:**
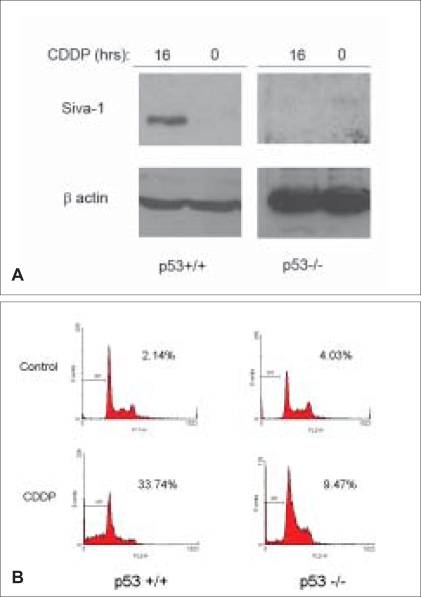
Cisplatin induces p53 dependent Siva-1 expression. HCT116 colon cancer p53+/+ and -/- cell lines (gift of Bert Vogelstein, Johns Hopkins University) were treated with DMSO (0, Control) or 100 μM cisplatin (CDDP, Sigma), incubated for 16 hours. (A) Endogenous Siva-1 expression by SDS-PAGE and immunoblotting of lysates with Siva-1 polyclonal antibody. beta-actin served as a loading control. (B) Apoptosis by propidium iodide staining. Percentages indicate sub-G1 apoptotic DNA analyzed by flow cytometry (FACScaliber, BD Biosciences)

To determine the significance of p53 expression on the pro-apoptotic function of Siva-1, we took advantage of p53+/+ and p53-/- HCT116 cells in our studies. Cells of both genotypes were treated with cisplatin (100 μM) or DMSO for 16 hours and endogenous Siva-1 levels were analyzed by immunoblotting whole cell lysates with Siva-1 polyclonal antibody, as described previously.[14] While we observed a robust induction of Siva-1 in response to cisplatin treatment in p53+/+ cells, we were unable to detect the protein in p53-/-cells (as compared to a relatively strong expression of beta-actin, used as a loading control) [Figure 1A]. To ensure that the cisplatin dose was sufficient to induce apoptosis, HCT116 cells were analyzed by propidium iodide staining. Significant levels of sub-G1 apoptotic DNA were observed in p53+/+ cells (increase from 2.14 to 33.74%); conversely, as expected, p53-/- cells exhibited levels of apoptosis only slightly higher than that of control treated cells (4.03 to 9.47%) [Figure 1B].

**Figure 2:**
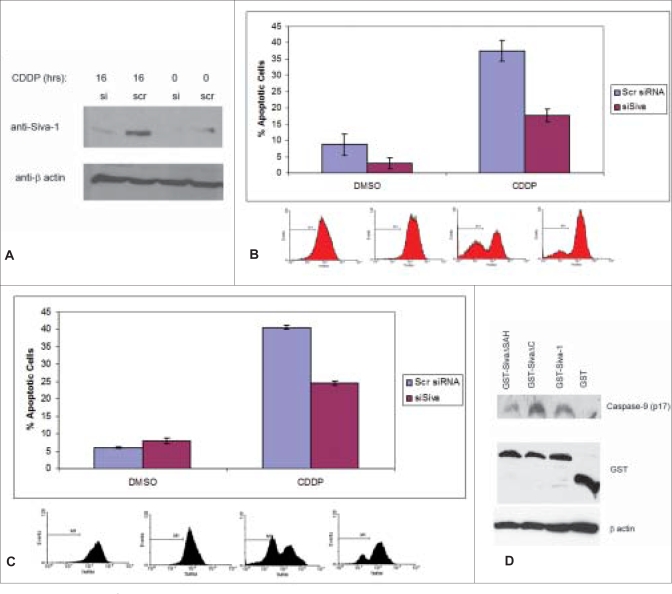
Abrogation of Siva-1 expression significantly impairs cisplatin-mediated apoptosis. (A) HCT116 p53+/+ or (C) Jurkat cells were transduced with lentiviruses encoding either Siva siRNA (siSiva) or scrambled control (Scr siRNA). Transduced cells were analyzed for Siva-1 expression (A). Percentage apoptosis in HCT116 p53+/+ (B) and Jurkat cells (C) are shown (D) HCT116 p53-/- cells were transfected with plasmids expressing GST, GST-Siva-1, GST-Siva-1ΔSAH or GST-Siva-1ΔC (residues 130-149) as described earlier.[8] The cell lysates were used to determine active caspase-9

In order to define the role of endogenous Siva-1 in DNA damage-induced apoptosis and its relationship to p53, we generated Siva-1 knockdown p53+/+ HCT116 cells using Siva-1 specific siRNA. The cells were transduced with recombinant lentiviruses encoding either unique Siva-1 siRNA (siSiva) or a corresponding scrambled control siRNA, as described by Gudi *et al*.[10] Since Siva-1 protein expression was not detectable in untreated cells [Figures 1A, 2A], we tested the effect of cisplatin treatment on apoptosis in siSiva and control cells. As shown in Figure 2A, cisplatin treatment induced significant Siva-1 expression in HCT116 p53+/+ cells that were dramatically reduced by prior transduction with recombinant lentivirus, capable of expressing siSiva.

Siva-1 plays an essential but partial role in DNA damage-induced apoptosis.

We next treated both Siva-1 knockdown and control HCT116 p53+/+ cells for 24 hours, with either cisplatin or DMSO. Apoptotic cells were quantified by flow cytometry using TMRM staining, to monitor loss of mitochondrial membrane potential. While control siRNA treated cells demonstrated a strong apoptotic response to cisplatin (about five-fold increase over mock treated cells), the percentage of programmed cell death in siSiva cells decreased by more than 50% and the difference was statistically significant at *P*<0.01 [Figure 2B]. To ensure that this effect was not cell type specific, Jurkat T cells were also similarly treated. As shown, a significantly diminished level of apoptosis was observed in siSiva cells treated with cisplatin [Figure 2C]. These findings clearly identify an essential but partial role for endogenous Siva-1 in cancer cell apoptosis engendered by cisplatin-induced terminal DNA damage.

### Siva-1 induces Bax oligomerization

In addition to its transcriptional role, p53 can function directly at the mitochondrial level and activate Bax, via a specific interaction with Bcl-x_L_.[15] In order to assess whether Siva-1 requires cytoplasmic p53 for its apoptotic function, we expressed Siva-1 in HCT116 p53-/- cells. Cells were transiently transfected with a GST-tagged Siva-1 or GST alone, incubated for 24 hours, and treated with cisplatin (100 μM) for a further 24 hours. Representative lysates were analyzed by immunoblotting for caspase-9 processing, a hallmark of activation of the intrinsic cell death pathway. As shown, a distinct caspas-9 related p17 cleavage product was visible in the lysates from Siva-1 expressing cells exposed to cisplatin; conversely, we were unable to detect this product in the case of cells expressing GST alone [Figure 2D, upper panel]. Although the relative expression of Siva-1 was considerably less than that of GST, it significantly enhanced cisplatin-induced apoptosis [Figure 2D, middle panel]. As expected, both the mutants migrated to a similar distance as that of the GST-Siva-1 WT on a 10% SDS-PAGE, since the difference in molecular weights was relatively small. We further determined that in contrast to a corresponding control deletion mutant (ΔC), a GST-Siva-1 mutant, lacking the SAH region, was deficient in its ability to promote caspase-9 activation [Figure 2D, upper panel].

### The Siva-1 SAH peptide can induce cytochrome c leakage in isolated mitochondrial fraction

**Figure 3:**
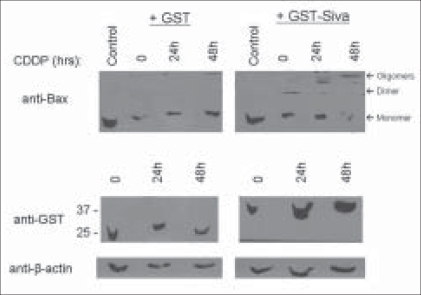
Siva-1 promotes Bax activation in response to cisplatin treatment. MDA-MB-231 cells transiently expressing GST or GST-Siva-1 were treated with DMSO or cisplatin (50 μM) for the indicated time points (upper panels). Cells were iterated with BMH cross-linker (Pierce) to observe Bax multimers; arrows indicate monomeric, dimeric, and oligomeric forms (upper panels). In a parallel experiment, appropriate expression levels and protein loading were confirmed using GST and and #946; actin antibodies, respectively (lower panels)

Intrinsic apoptosis mediated by p53-induced apoptotic genes is characterized by the oligomerization of Bax and Bak whose function is normally curtailed by anti-apoptotic members such as Bcl-2 and Bcl-x_L_. Activated Bax oligomerizes and inserts into the mitochondrial membrane, thereby forming channels through which resident pro-apoptotic proteins such as cytochrome C leak into cytosol.[21] We made use of a chemical cross-linker (BMH) to observe such potential higher order Bax structures, as an indicator for intrinsic apoptosis.[22] MDA-MB-231 cells, known to lack functional p53, were transfected with either GST or GST-Siva-1 expressing plasmids; following a 24-hour expression period, cells were treated with suboptimal doses of cisplatin[13] or DMSO for 24-48 hours. Harvested lysates were then incubated with BMH, prior to immunoblotting, to covalently crosslink proteins. Intriguingly, in GST-Siva-1 expressing cells, Bax appeared to dimerize, even in the absence cisplatin treatment; following 24 and 48 hours of drug exposure, oligomeric complexes consistent with Bax trimers and tetramers, respectively, were observed, suggesting a clear progression in Bax activation over the experimental time period [Figure 3, upper right panel]. However, a vast majority of the Bax protein appeared to remain in a monomeric state in cells expressing GST, regardless of the duration of cisplatin treatment [Figure 3, upper left panel]. Expression levels of GST and GST-Siva-1 were determined and the specificity of the result upheld [Figure 3, lower panels].

**Figure 4:**
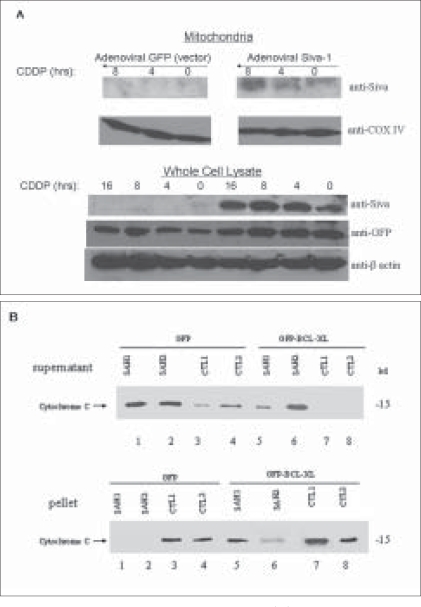
Siva-1 and cytochrome C release. (A) HCT116 p53-/- cells were infected with either Siva-1 (Adeno-Siva-1) or GFP (Adeno-GFP) and treated as shown. Heavy membrane fraction lysates were analyzed for Siva-1 and COX IV expression (upper panels); corresponding whole cell lysate samples were analyzed for Siva-1 and GFP expression levels (lower panel). (B) Heavy membrane fractions (30 μg) from MCF-7 cells stably expressing either GFP or GFP-Bcl-xL[8] were incubated with either 5 or 10 μg of SAH or control peptides. At 30 minutes, the fractions were analyzed for cytochrome C

Since proapoptotic BH3-only Bcl-2 family members function principally at the mitochondrial level, we next investigated the intracellular localization pattern of Siva-1. HCT116 p53-/- cells were transduced with either Siva-1 or control IRES-GFP expressing recombinant adenoviruses; following 24 hours of expression, cells were treated with cisplatin and subjected to hypotonic shock. They were then fractionated to obtain heavy membrane (mitochondria-enriched) isolates. Biochemical analysis of cells treated with cisplatin over a 4-8 hour period revealed incremental accumulation of Siva-1 in the mitochondrial compartment [Figure 4A, upper right panel]. As observed previously, endogenous Siva-1 levels could not be detected in the p53 -/- cell line [Figure 4A, upper left panel]. Whole cell lysate immunoblot analysis confirmed specific and appropriate viral transduction and Siva-1 expression [Figure 4A, lower panels]. Having verified mitochondrial localization of Siva-1 in response to genotoxic stress and its independence of cytoplasmic p53, we further investigated the potential mechanistic consequences of Siva-1 at the mitochondrial level.

We have previously demonstrated that sole expression of the SAH domain of Siva is sufficient to negate the protective effects of Bcl-x_L_ and Bcl-2 in response to DNA damage, resulting in cytochrome C release and consequent caspase-9 activation.[8–9,13] To investigate the functional relevance of current translocation data, isolated heavy membrane fractions from MCF-7 cells stably expressing GFP or a GFP-tagged version of Bcl-x_L_ were incubated with either 5 [Figure 4B: SAH1, CTL1] or 10 (SAH2, CTL2) µg of synthetic peptides, corresponding to the SAH region of Siva-1 (amino acid residues 36-55) or a control downstream region (amino acid residues 130-149). Equivalent mitochondria (pellet) and corresponding supernatant fractions were then assessed for cytochrome C by western blot. As shown, mitochondria from GFP expressing cells exhibited nearly complete efflux of cytochrome C from pellet fractions, when treated with either concentration of SAH peptide; this corresponded with reciprocal accumulation in the supernatant. Mitochondria treated with either concentration of control peptide demonstrated comparatively less cytochrome C release [Figure 4B, left panels]. Interestingly, a similar effect was observed when mitochondria from cells stably expressing GFP-Bcl-x_L_ were treated with SAH peptide. Notably, however, higher peptide concentrations were required for significantly enhanced cytochrome C efflux. This is likely due to enhanced levels of expression of Bcl-x_L_ in transfected cells, which necessitated higher quantities of SAH peptide to overcome the protective effect of Bcl-x_L._ Mitochondria treated with control peptide elicited little or no effect [Figure 4A, right panels]. This suggests that the SAH region of Siva is both necessary and sufficient to evoke cytochrome C release from mitochondria, reminiscent of a BH3 domain-like mechanism, and it supports previous and current data demonstrating SAH dependent intrinsic apoptotic initiation via caspase-9 [Figure 2D].

## Discussion

In this paper, we have shown that Siva-1, a pro-apoptotic protein that lacks a true BH3 domain, behaves like a small BH3 only protein and plays a significant and essential but partial role in genotoxic (p53-induced) apoptosis. Using the colorectal cancer cell line HCT116, we have clearly shown the dependence of Siva-1 intracellular expression on functional p53. The expressed Siva-1 targets mitochondria and induces Bax oligomerization and caspase-9 activation and the twenty amino acid putative helical region in Siva-1 (SAH) is the principal mediator.

Our data demonstrating the dependence of endogenous Siva-1 expression on induced p53 activity is in accordance with previous reports published by Pommmier and coworker (2003) using gene profiling. They showed that the topoisomerase inhibitor topotecan induces Siva-1 expression (transcripts) in a p53 dependent manner.[12] A previous study of differential gene expression analysis using cell cycle and apoptosis DNA microarray in cisplatin treated Hep3B cells also reveals significant induction of Siva expression.[11] In addition, our data is also in agreement with a report previously published by Slack and coworkers that p53 as well as E2F1 directly activate the transcription of Siva-1.[4] It is therefore not surprising that p53, a powerful tumor suppressor, induces apoptosis in susceptible cells by upregulating transcription of the pro-apoptotic gene Siva-1. As shown in Figure 2A, Siva-1 knockdown alone appears to promote cell survival; however, we have observed this only in HCT116 but not in Jurkat cells and, therefore, disregarded the observation. The maximum effect of Siva-1, however, was seen in cells treated with various apoptotic stimuli.[7–10,13]

Although it lacks significant homology to the Bcl-2 family *per se*, we have shown here that the ability of Siva-1 to bind anti-apoptotic proteins like Bcl-x_L_ may consequently result in direct activation of Bax and cytochrome C release. The unique putative twenty amino acid SAH region in Siva-1 was shown by us to be essential for its apoptotic function. Based on our earlier observation,[10] it appears to behave like the BH3 only peptide of Bid, in inducing apoptosis.[16] The time dependent increase in expressed Siva-1 in heavy membrane fraction, upon cisplatin treatment, is highly likely to be due its localization to mitochondria, Here we have shown that treatment of mitochondrial fraction with the above peptide results in cytochrome C leakage; in our previous studies, we demonstrated that it can specifically interact with Bcl2 or Bcl-xl but not Bax.[13] Thus, induction of Siva-1 by p53 (genotoxic stress) is likely to result in its translocation to mitochondria and neutralize the pro-survival effects of Bcl2 anti-apoptotic members. This explains the enhanced Bax oligomerization seen in cells forced to overexpress Siva-1 [Figure 3].

A large body of work supports the idea that the antiapoptotic members such as Bcl2 and Bcl-xL counteract the apoptotic effects of Bax and Bak and thus act as sentinels of cell survival. Under conditions where the DNA is damaged and irreparable (genotoxic stress), the relatively small proapoptotic molecules such as Bid, Bad and Puma interfere with the above interaction, resulting in the release of Bax and Bak and their oligomerization. Oligomerization of Bax and Bak, in turn, results in loss in mitochondrial integrity and leakage of cytochrome c. The above events are typically induced by small BH3 only apoptotic molecules that are induced by p53. Siva-1, although induced by p53, is not a member of the Bcl2 family and yet induces oligomerization of Bax.

P53 is known to induce intrinsic apoptosis in a transcriptional independent as well as transcriptional dependent manner. The most well characterized proapoptotic proteins that are transcriptionally regulated by p53 are Bax and the BH3 only protein Puma.[17] In normal cell, the levels of p53 are very low; however, they are rapidly elevated upon induction of DNA damage. This results in accumulation of p53 in cytosol. The apoptotic activity of p53 is neutralized by Bcl2 and Bcl-xl. Under the above conditions, Puma levels are also known to increase. It has a high affinity for Bcl2 and Bcl-xL and, thus, displaces p53 from Bcl2 and Bcl-xl, resulting in intrinsic apoptosis.[18] There appears to be a strong dependence of Puma for p53, since it fails to induce significant apoptosis in p53 null cells.[2,18] In this sense, Siva-1 appears to be different from that of Puma. Its ability to promote intrinsic cell death does not require p53, since expression of Siva-1 in p53 null cells also enhances cisplatin-induced cell death [Figure 2D].[13] Since elimination of DNA damaged cells to prevent tumorigenesis is a very important function, it appears to be regulated by redundant and overlapping pathways. For instance, p53 regulated Noxa appears to have a definitive role in ROS-induced cell death, whereas Puma is an essential mediator of DNA damage-induced cell death. The role of Puma appears to be somewhat limited by functional p53, although its transcription can also be regulated by E2F1.

Recently, an essential role for Siva-1 was also shown in campotheticin-induced apoptosis in cerebellar neuronal granules, that was also p53 dependent.[23] The fact that expression of Siva-1 can potentiate cisplatin-induced apoptosis even in the absence of a functional p53 means that Siva-1 is likely to play a role in genotoxic stress-induced apoptosis, even if p53 is mutated and non-functional, as is the case with almost 50% of cancers.

Although we have shown that Siva-1 plays an essential and significant role in cisplatin-induced intrinsic cell death, despite the knockdown of Siva-1 expression, the cells still undergo considerable apoptosis. This could be explained by the fact that p53 induces transcription of several apoptotic genes, other than Siva-1, and these could be responsible for the residual cell death.[17]

## Conclusions

**Figure 5:**
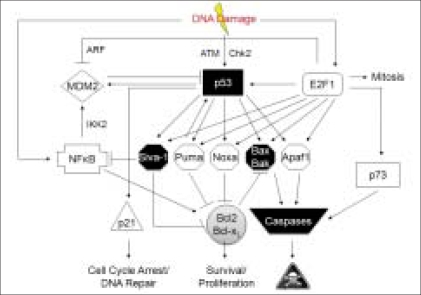
Siva-1 plays a necessary but partial role in genotoxicinduced apoptosis; p53 is a central player in DNA damage-induced apoptosis and it triggers transcription of several pro-apoptotic genes such as Siva-1, Puma, Noxa, Bax etc. Siva-1, though principally regulated by p53 transcriptional activity, can function independent of p53 (unlike Puma), whereas Noxa appears to be important for mediation of ROS-induced cell death. Finally, the apoptosis pathways are kept in check by cell survival pathways, wherein Siva-1 is known to inhibit NF-kappa B.[10]

We have attempted to depict the complex regulation of DNA damage-induced apoptosis in the model shown in Figure 5. There is no doubt that p53 is a central player recruited by the cell, in response to DNA damage, and it decides the fate of the cell mainly by switching on specific gene transcription programs. By itself and by positively increasing the transcription of several apoptotic genes, it triggers intrinsic cell death. Siva-1 is one of the effectors of p53; however, its transcription can also be triggered by other transcription factors such as E2F1. Unlike Puma, Siva-1 mediates cell death in a p53 independent manner. The fact that Siva-1 can also potentially inhibit NF-kappa B activation that promotes transcription of several cell survival molecules[10] also makes this a unique effector of p53 mediated apoptosis.
